# Association between tumor volume changes during neoadjuvant chemoimmunotherapy and survival outcomes in esophageal squamous cell carcinoma: a multicenter study

**DOI:** 10.3389/fimmu.2026.1772722

**Published:** 2026-04-14

**Authors:** Hao Zhou, Bo Li, Shuai Wang

**Affiliations:** 1 Department of Radiation Oncology, Shandong Cancer Hospital and Institute, Shandong First Medical University and Shandong Academy of Medical Sciences, Jinan, Shandong, China; 2 Department of Radiation Oncology, Yantai Yuhuangding Hospital, Qingdao University, Yantai, China

**Keywords:** chemotherapy, computed tomography, esophageal cancer, immunotherapy, timing of surgery

## Abstract

**Purpose:**

In recent years, neoadjuvant chemoimmunotherapy (NICT) has become a research focus in the treatment of esophageal cancer. This study aims to evaluate the predictive value of tumor volume changes before and after NICT for prognosis in patients with operable esophageal squamous cell carcinoma (ESCC).

**Materials and methods:**

This retrospective study included 163 patients with histologically confirmed ESCC from two medical centers between October 1, 2020, and October 1, 2022. All patients received NICT before undergoing radical esophagectomy. Based on pre- and post−treatment CT images, we delineated and calculated the volume of the esophageal tumor. The rate of tumor volume change was then analyzed for its association with patient prognosis. The study endpoints were overall survival (OS), disease−free survival (DFS) and progression−free survival (PFS).

**Results:**

Multivariate regression analysis indicated that tumor location, number of NICT cycles, and tumor volume change rate were independent influencing factors for both DFS and PFS. The interval between NICT completion and surgery, along with tumor volume change rate, were independent factors for OS. Significant tumor volume reduction served as a predictor of favorable prognosis, demonstrating certain predictive value for long-term survival in patients. Surgery performed within 6 weeks of neoadjuvant therapy was significantly associated with longer OS, while DFS and PFS also showed a trend toward improvement.

**Conclusion:**

The change in tumor volume before and after neoadjuvant therapy is an independent prognostic factor for esophageal cancer patients receiving NICT. Additionally, performing radical surgery as soon as possible after completing neoadjuvant therapy is associated with improved survival outcomes.

## Introduction

Esophageal cancer (EC) ranks as the eighth most commonly diagnosed malignancy and the sixth leading cause of cancer-related death worldwide, posing a significant public health challenge (1). The two main pathological types of the disease are adenocarcinoma and esophageal squamous cell carcinoma (ESCC), with the latter being particularly prevalent in Asian populations (2). Despite advances in multidisciplinary treatment strategies centered on surgery and radiotherapy, the overall prognosis for patients with esophageal cancer remains suboptimal. Thus, optimizing therapeutic approaches continues to be a central issue in clinical research (3–5).

As a critical step prior to radical surgery, neoadjuvant therapy aims to reduce tumor size, increase the R0 resection rate, and eliminate micro metastases, thereby lowering the risk of postoperative recurrence (6, 7). For locally advanced, resectable esophageal cancer, neoadjuvant chemoradiotherapy (NCRT) has emerged as the standard of care (8, 9). While NCRT improves treatment efficacy, it is also associated with an increased risk of postoperative complications and mortality, which limits its application in certain patient populations (10, 11). Consequently, the development of effective but with controllable toxicity neoadjuvant regimens represents a critical unmet need in current clinical practice (12).

In recent years, NICT has emerged as an innovative strategy, demonstrating significant potential in the treatment of esophageal cancer (13, 14). Immune checkpoint inhibitors (ICIs) work by blocking signaling pathways such as PD-1/PD-L1, thereby restoring the immune system’s ability to attack tumor cells. When combined with chemotherapy, they not only enhance the anti-tumor immune response but also effectively reverse the immunosuppressive tumor microenvironment (15, 16). Following confirmed efficacy and safety in widespread clinical use, ICIs including camrelizumab and pembrolizumab are now guideline-recommended for esophageal cancer in the first-line, second-line, and postoperative adjuvant settings (17–19). Compared with NCRT, NICT avoids radiation-related toxicities. This simplification of the treatment pathway facilitates earlier radical surgery and reduces procedural complexity, thereby enhancing treatment compliance and overall therapeutic efficiency (20–22). Studies have confirmed that NICT demonstrates favorable efficacy and safety in patients with locally advanced ESCC, without significantly increasing surgical risks (14, 23). It thus offers a new therapeutic option and hope for improved survival in this patient population.

Computed tomography (CT) is the most widely used imaging modality for evaluating treatment response in solid tumors. Dynamic changes in tumor volume measured by CT have been validated to predict long−term prognosis in multiple solid tumors. Studies have shown that primary tumor volume is closely associated with disease progression and survival outcomes (24, 25). For instance, in esophageal cancer patients undergoing definitive concurrent chemoradiotherapy, the extent of tumor volume reduction during treatment has been established as a reliable prognostic factor (25). However, in the increasingly emphasized field of NICT, the prognostic significance of tumor volume changes remains unclear, and reliable biomarkers for evaluating the efficacy and prognosis of NICT have yet to be established. As a non-invasive and repeatable imaging indicator, the systematic assessment of tumor volume changes during NICT holds promise for providing a crucial basis for personalized treatment and prognostic judgment in patients with esophageal cancer.

Therefore, this study aims to investigate the relationship between tumor volume changes and prognosis in esophageal cancer patients receiving NICT. By analyzing tumor response on pre-operative CT images, we seek to enable an early and objective assessment of postoperative progression and long-term survival.

## Materials and methods

### Patients

This study retrospectively enrolled 163 patients with esophageal cancer from Shandong Cancer Hospital and Yantai Yuhuangding Hospital between October 1, 2020, and October 1, 2022. All patients were newly diagnosed and histologically confirmed cases. Prior to enrollment, each patient underwent a comprehensive imaging evaluation, including barium esophagography, endoscopic examination, and CT scans.

The inclusion and exclusion criteria were as follows:

Inclusion Criteria: Age 18–75 years; Cytologically or histopathologically confirmed esophageal squamous cell carcinoma (ESCC); Received at least two cycles of chemoimmunotherapy; Clinical stage T1N+M0 or T2–4NanyM0, classified as resectable locally advanced disease according to the UICC/AJCC 9th edition TNM staging system, with no clinical or radiographic evidence of distant metastasis; Underwent radical en bloc resection with achieved R0 resection (negative distal, proximal, and circumferential margins) following NICT.

Exclusion Criteria: Presence of other primary malignancies; Incomplete clinical or imaging data; Any history of prior antitumor therapy.

### Treatment plan

Following comprehensive evaluation, all patients were deemed eligible for radical esophagectomy. Preoperative assessment included pulmonary function tests, electrocardiography, echocardiography, chest CT, and laboratory hematological tests to confirm surgical feasibility. The neoadjuvant chemotherapy regimen was platinum−based, combined with either fluorouracil (PF regimen) or paclitaxel/albumin−bound paclitaxel (TP regimen). Drug doses were individually adjusted based on each patient’s clinical condition. The immunotherapy regimen consisted of intravenous infusion of a programmed death-1 (PD-1) inhibitor, such as camrelizumab, sintilimab, or toripalimab. Approximately 4 to 8 weeks after completing neoadjuvant therapy, patients underwent radical esophagectomy and/or systematic lymphadenectomy under general anesthesia.

### Imaging examination and efficacy evaluation

All patients underwent contrast-enhanced chest CT scans before treatment initiation and within 2–3 weeks after completing neoadjuvant therapy. Scans were performed using a Philips 16-slice large-bore CT scanner (Philips Healthcare, Amsterdam, The Netherlands) with a slice thickness of 3 mm. Images were acquired in the supine position after intravenous administration of iodine-based contrast agent, covering a range from the lower neck to the upper abdomen. Axial images were reconstructed in three dimensions for analysis. Tumor volume was manually delineated on the enhanced CT images by a senior radiation oncologist, with reference to barium esophagography, PET/CT, and endoscopic ultrasound. The regions of interest included areas of eccentric or circumferential esophageal wall thickening exceeding 5 mm (excluding the lumen) (25). For each patient, tumor volume delineation before and after treatment was performed by the same physician to ensure Intrapatient consistency in longitudinal data and to minimize measurement errors arising from inter-observer variability. All image delineations were conducted jointly by two radiation oncologists, each with over ten years of clinical experience, who strictly adhered to a standardized delineation protocol. Subsequently, the tumor volume was automatically calculated based on the 3D Slicer system, and the tumor volume change rate was further derived using the following formula: 100% × (post-treatment volume − pre-treatment volume)/pre-treatment volume. To assess inter- and intra-observer reliability of tumor volume delineation, 15 patients were randomly selected from each investigator’s patient cohort for a supplementary analysis. Inter-observer reliability was evaluated using a crossover design: the two investigators independently performed blinded delineations on cases originally processed by the other. Intra-observer reliability was assessed using a test-retest design: two months after the initial delineation, both investigators performed a second blinded delineation on the same set of cases they had previously managed. A two-way random-effects model with absolute agreement was used to calculate the intraclass correlation coefficient (ICC) and its 95% confidence interval (CI) to evaluate reproducibility. The results demonstrated excellent reliability, with an inter-observer ICC of 0.939 (95% CI: 0.874–0.971) and an intra-observer ICC of 0.959 (95% CI: 0.914–0.981). Follow-up commenced one month after treatment completion, scheduled every 3 months for the first 3 years and every 6 months thereafter until patient death. Assessments included CT, esophagography, and endoscopy. Pathological complete response (pCR) was defined as ypT0N0, indicating the absence of viable tumor cells in both the primary site and lymph nodes. Tumor regression grade (TRG) was assessed using a four-tier system: TRG0 (complete response, no residual cancer cells), TRG1 (moderate response, only small clusters or single cancer cells), TRG2 (mild response, residual cancer with predominant fibrosis), and TRG3 (poor or no response, minimal necrosis with extensive residual cancer) (26).

### Statistical analysis

In this study, the primary endpoints were defined as follows: Disease-Free Survival (DFS) was defined as the time from the date of surgery to disease progression or death from any cause; Progression-Free Survival (PFS) was defined as the time from the initiation of neoadjuvant therapy to disease progression or death from any cause. These two endpoints are complementary: whereas PFS starts at treatment initiation and spans the entire course to link early radiological response with overall disease control, DFS focuses on the postoperative disease-free state as a classic indicator of long-term outcomes. The combination of these two endpoints aims to systematically validate the prognostic value of tumor volume change rates across different disease stages. Overall Survival (OS) was defined as the time from the initiation of neoadjuvant therapy to death from any cause. Continuous variables were compared with the independent t−test or Mann−Whitney U test, and categorical variables with the χ² or Fisher’s exact test. First, univariate Cox regression identified variables with P < 0.05, which were then entered into a multivariate Cox model to obtain hazard ratios (HRs) and 95% CIs. Spearman’s rank correlation analysis was employed to assess correlations among variables; stepwise regression was then used for variable selection to mitigate multicollinearity, with a variance inflation factor (VIF) threshold of < 5. Given that tumor volume change rate served as the key prognostic indicator in the present study, it was forced into the multivariable model. Survival curves were plotted by the Kaplan−Meier method and compared with the log−rank test. Prognostic discriminatory performance assessed by time-dependent ROC curve analysis. Propensity score matching (PSM) was employed to balance baseline covariates. The matching was performed using the nearest-neighbor method with a caliper width of 0.2. The matching ratio (1:1 or 1:N) was determined based on the relative size of the patient cohorts. After matching, intergroup balance was assessed by calculating the standardized mean difference (SMD). An absolute SMD value < 0.1 was considered to indicate excellent balance, a value between 0.1 and 0.2 suggested acceptable balance, and a value > 0.2 indicated a potentially substantial residual imbalance. A P value < 0.05 was considered significant. All analyses used SPSS (v27.0) and R (v4.5.0).

## Results

### Patient

This study ultimately included 163 patients. Baseline characteristics showed a predominance of male patients (140, 85.9%), with a median age of 65 years. The T3 stage was the most common pathological stage (118, 72.4%). All patients successfully completed NICT, of whom 44 (27.0%) received more than two cycles. The median follow-up time was 41.1 months. Radiographic assessment revealed that the vast majority of patients (151, 92.6%) achieved tumor volume reduction after NICT, while only 12 (7.4%) showed a slight increase; the overall median tumor volume change rate was −50.30%. Among these 12 patients with slight primary tumor enlargement, the postoperative pathological regression grades (TRG) were distributed as follows: 2 patients (16.7%) achieved pathological complete response (pCR, TRG 0), 3 (25.0%) had TRG 1, 3 (25.0%) had TRG 2, and 4 (33.3%) had TRG 3. Pathological analysis indicated a pCR rate of 31.3% for esophageal squamous cell carcinoma. For the entire cohort, the 2- and 3-year DFS rates were 69.9% and 61.3%, the 2- and 3-year PFS rates were 74.2% and 62.0%, and the 2- and 3-year OS rates were 87.1% and 79.1%, respectively. Among the 68 patients who experienced disease progression, 32 (47.1%) developed local recurrence, 22 (32.4%) developed distant metastasis, and 14 (20.6%) presented with both.

### Analysis of associations between clinical and volumetric features and prognosis

**Figure 1:**
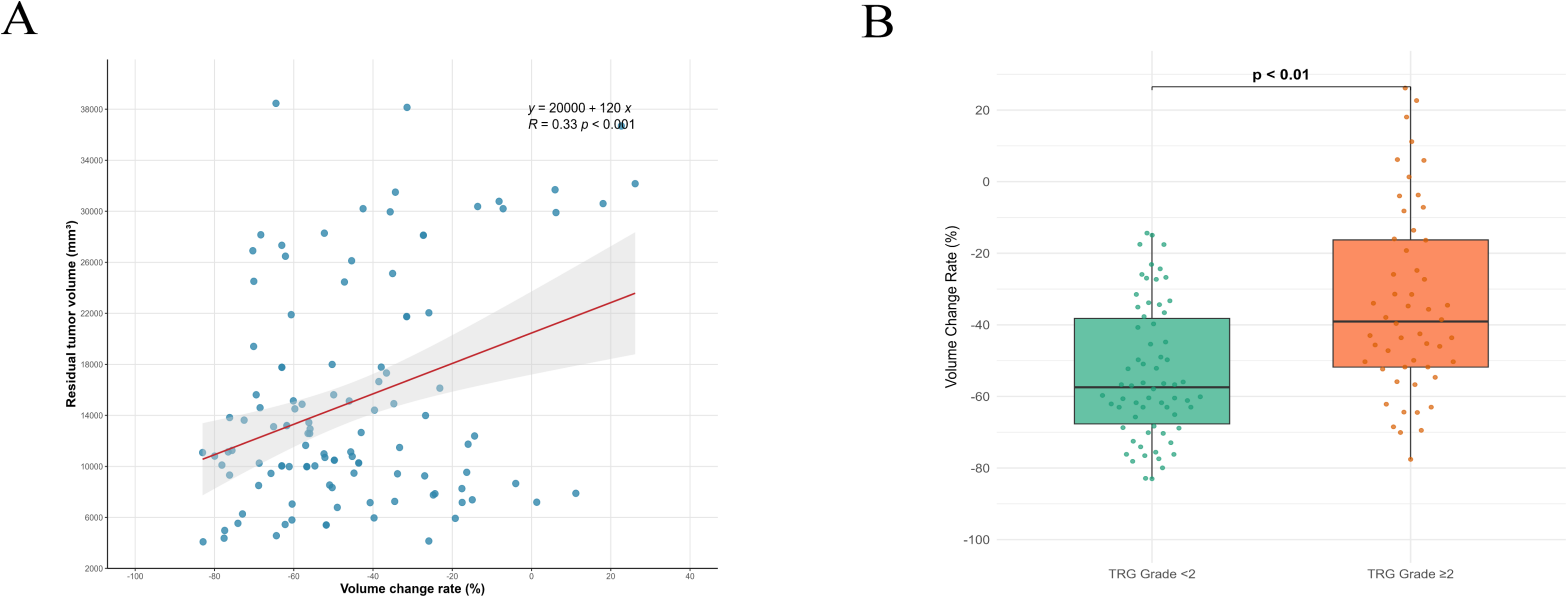
**(A)** Association between residual tumor volume and volume change rate. **(B)** Association between TRG and volume change rate.

**Figure 2:**
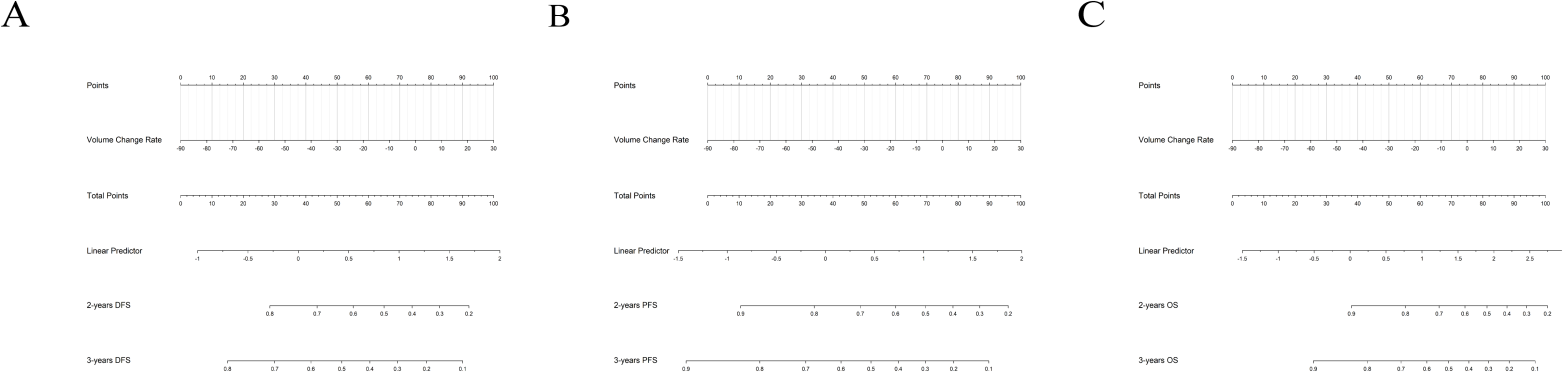
**(A)** DFS nomogram based on volume change rate. **(B)** PFS nomogram based on volume change rate. **(C)** OS nomogram based on volume change rate.

**Figure 3:**
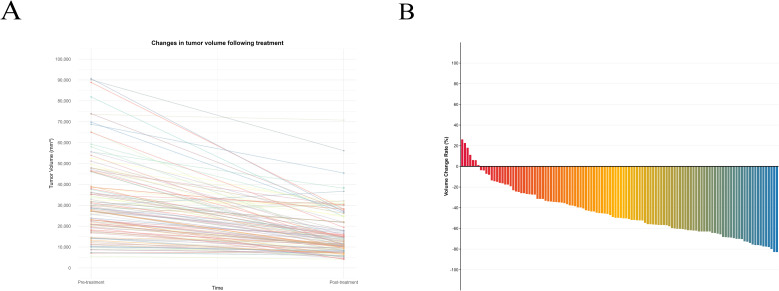
**(A)** Line chart of tumor volume changes in patients before and after treatment. **(B)** Histogram of tumor volume change rate.

First, we compared the baseline characteristics between Shandong Cancer Hospital (n=118) and Yantai Yuhuangding Hospital (n=45). The statistical results showed no significant differences between the two groups (**Table 1**). Subsequently, the 118 patients from Shandong Cancer Hospital served as the initial cohort for further analysis. **Table 2**, **3** and **4** summarize the clinical characteristics, tumor volume parameters, and the results of univariate and multivariate analyses in relation to DFS, PFS, and OS. Multivariate Cox analysis identified tumor location, number of NICT cycles, and the tumor volume change rate as independent factors for both DFS and PFS. For OS, the interval from NICT completion to surgery and the tumor volume change rate were independent prognostic factors. Correlation analysis revealed a significant positive correlation between the rate of tumor volume change and residual tumor volume (P < 0.001). Additionally, patients with TRG < 2 exhibited a significantly lower rate of tumor volume change compared to those with TRG ≥ 2 (P < 0.001) (**Figure 1**). Further analysis confirmed a significant positive correlation between TRG and the rate of tumor volume change (r = 0.425, P < 0.001). Based on these findings, we developed a prognostic nomogram incorporating the volume change rate (**Figure 2**). A spaghetti plot and a waterfall plot were created to visually depict the dynamic changes in tumor volumes before and after treatment (**Figure 3**).

**Table 1:** Baseline characteristics of patients.

Characteristics	Training set (n=118)	Validation set (n=45)	χ²	*p*
Sex				
Female	14 (11.9)	9 (20.0)	1.779	0.182
Male	104 (88.1)	36 (80.0)		
Age				
< 60	49 (41.5)	12 (26.7)	3.071	0.080
≥ 60	69 (58.5)	33 (73.3)		
KPS				
< 90	94 (79.7)	33 (73.3)	0.758	0.384
≥ 90	24 (20.3)	12 (26.7)		
Tumor Location				
Upper	21 (17.8)	7 (15.6)	0.115	0.735
Middle and Lower	97 (82.2)	38 (84.4)		
T stage				
≤ 2	11 (9.3)	5 (11.1)	0.118	0.731
> 2	107 (90.7)	40 (88.9)		
N stage				
< 2	88 (74.6)	30 (66.7)	1.020	0.313
≥ 2	30 (25.4)	15 (33.3)		
Time Interval				
≤ 6 weeks	93 (78.8)	31 (68.9)	1.763	0.184
> 6 weeks	25 (21.2)	14 (31.1)		
TRG				
0	39 (33.1)	12 (26.7)	2.164	0.539
1	27 (22.9)	8 (17.8)		
2	35 (29.7)	15 (33.3)		
3	17 (14.4)	10 (22.2)		
NICT cycles				
2 cycles	89 (75.4)	30 (66.7)	1.268	0.260
> 2 cycles	29 (24.6)	15 (33.3)		
Adjuvant therapy				
No adjuvant therapy	33(28.0)	10 (22.2)	-	0.799
Chemotherapy	17 (14.4)	6 (13.3)		
Chemoradiotherapy	17 (14.4)	10 (22.2)		
chemo-immunotherapy	41 (34.7)	16 (35.6)		
Chemoradiotherapy plus immunotherapy	10 (8.5)	3 (6.7)		
Volume Change Rate (%)	−49.84 (−63.00–−31.48)	−50.70 (−59.72–−21.83)	-	0.727
Residual Tumor Volume (mm^3^)	12481 (9304–21782)	14335 (9972–18644)	-	0.730

KPS, Karnofsky Performance Status; TRG, Tumor Regression Grade; NICT, Neoadjuvant Chemoimmunotherapy; Time Interval, Duration from NICT completion to surgery.

**Table 2:** Univariate and multivariate Cox proportional hazards regression analysis of factors influencing DFS.

Characteristics	Univariate Cox regression		Multivariable Cox regression	
	HR (95%CI)	*p*	HR (95%CI)	*p*
Sex				
Female	Ref			
Male	1.66 (0.60–4.62)	0.331		
Age				
< 60	Ref			
≥ 60	0.79 (0.45–1.39)	0.417		
KPS				
< 90	Ref			
≥ 90	0.73 (0.34–1.56)	0.414		
Tumor Location				
Upper	Ref		Ref	
Middle and Lower	0.44 (0.23–0.82)	0.011	0.43 (0.22–0.82)	0.011
T stage				
≤ 2	Ref			
> 2	2.72 (0.66–11.21)	0.166		
N stage				
< 2	Ref			
≥ 2	0.57 (0.28–1.18)	0.133		
Time Interval				
≤ 6 weeks	Ref			
> 6 weeks	1.13 (0.58–2.20)	0.730		
TRG				
0	Ref			
1	0.99 (0.38–2.59)	0.976		
2	2.92 (1.34–6.33)	0.007		
3	5.91 (2.60–13.40)	<0.001		
NICT cycles				
2 cycles	Ref		Ref	
> 2 cycles	1.98 (1.10–3.58)	0.023	2.29 (1.26–4.17)	0.007
Adjuvant therapy				
No adjuvant therapy	Ref			
Chemotherapy	0.78 (0.32–1.93)	0.597		
Chemoradiotherapy	0.93 (0.39–2.19)	0.862		
chemo-immunotherapy	0.58 (0.28–1.21)	0.146		
Chemoradiotherapy plus immunotherapy	1.13 (0.41–3.11)	0.811		
Volume Change Rate (%)	1.03 (1.01–1.04)	<0.001	1.03 (1.02–1.04)	<0.001
Residual Tumor Volume (mm^3^)	1.01 (1.01–1.01)	0.010		

KPS, Karnofsky Performance Status; TRG, Tumor Regression Grade; NICT, Neoadjuvant Chemoimmunotherapy; Time Interval, Duration from NICT completion to surgery.

**Table 3:** Univariate and multivariate Cox proportional hazards regression analysis of factors influencing PFS.

Characteristics	Univariate Cox regression		Multivariable Cox regression	
	HR (95%CI)	*p*	HR (95%CI)	*p*
Sex				
Female	Ref			
Male	1.64 (0.59–4.56)	0.344		
Age				
< 60	Ref			
≥ 60	0.80 (0.45–1.40)	0.431		
KPS				
< 90	Ref			
≥ 90	0.71 (0.33–1.52)	0.382		
Tumor Location				
Upper	Ref		Ref	
Middle and Lower	0.44 (0.23–0.83)	0.012	0.44 (0.23–0.85)	0.014
T stage				
≤ 2	Ref			
> 2	2.74 (0.66–11.27)	0.163		
N stage				
< 2	Ref			
≥ 2	0.58 (0.28–1.19)	0.135		
Time Interval				
≤ 6 weeks	Ref			
> 6 weeks	1.10 (0.56–2.16)	0.774		
TRG				
0	Ref			
1	0.99 (0.38–2.60)	0.983		
2	2.97 (1.37–6.44)	0.006		
3	5.98 (2.63–13.60)	<0.001		
NICT cycles				
2 cycles	Ref		Ref	
> 2 cycles	1.89 (1.05–3.41)	0.034	2.14 (1.18–3.88)	0.012
Adjuvant therapy				
No adjuvant therapy	Ref			
Chemotherapy	0.81 (0.33–1.98)	0.643		
Chemoradiotherapy	0.95 (0.40–2.25)	0.911		
chemo-immunotherapy	0.60 (0.29–1.24)	0.168		
Chemoradiotherapy plus immunotherapy	1.12 (0.41–3.08)	0.827		
Volume Change Rate (%)	1.03 (1.01–1.04)	<0.001	1.03 (1.02–1.04)	<0.001
Residual Tumor Volume (mm^3^)	1.01 (1.01–1.01)	0.010		

KPS, Karnofsky Performance Status; TRG, Tumor Regression Grade; NICT, Neoadjuvant Chemoimmunotherapy; Time Interval, Duration from NICT completion to surgery.

**Table 4:** Univariate and multivariate Cox proportional hazards regression analysis of factors influencing OS.

Characteristics	Univariate Cox regression		Multivariable Cox regression	
	HR (95%CI)	*p*	HR (95%CI)	*p*
Sex				
Female	Ref			
Male	4.20 (0.57–30.89)	0.159		
Age				
< 60	Ref			
≥ 60	0.69 (0.33–1.43)	0.322		
KPS				
< 90	Ref			
≥ 90	0.59 (0.21–1.70)	0.330		
Tumor Location				
Upper	Ref			
Middle and Lower	0.58 (0.25–1.36)	0.214		
T stage				
≤ 2	Ref			
> 2	1.38 (0.33–5.83)	0.657		
N stage				
< 2	Ref			
≥ 2	0.58 (0.22–1.52)	0.265		
Time Interval				
≤ 6 weeks	Ref			
> 6 weeks	2.22 (1.03–4.78)	0.041	2.34 (1.08–5.07)	0.031
TRG				
0	Ref			
1	0.71 (0.18–2.83)	0.625		
2	1.95 (0.69–5.48)	0.206		
3	7.10 (2.61–19.33)	<0.001		
NICT cycles				
2 cycles	Ref			
> 2 cycles	1.76 (0.82–3.79)	0.148		
Adjuvant therapy				
No adjuvant therapy	Ref			
Chemotherapy	0.29 (0.06–1.31)	0.108		
Chemoradiotherapy	0.85 (0.30–2.46)	0.769		
chemo-immunotherapy	0.42 (0.16–1.10)	0.077		
Chemoradiotherapy plus immunotherapy	1.30 (0.42–4.10)	0.649		
Volume Change Rate (%)	1.04 (1.02–1.05)	<0.001	1.04 (1.02–1.05)	<0.001
Residual Tumor Volume (mm^3^)	1.00 (1.00–1.00)	0.094		

KPS, Karnofsky Performance Status; TRG, Tumor Regression Grade; NICT, Neoadjuvant Chemoimmunotherapy; Time Interval, Duration from NICT completion to surgery.

**Figure 4:**
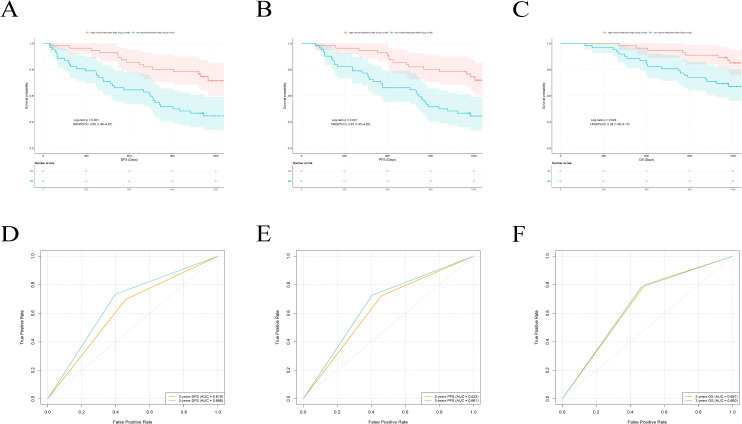
Kaplan-Meier survival analysis and ROC curve evaluation using a single-center dataset. **(A)** Kaplan-Meier analysis of DFS in patients stratified by high and low tumor volume change rate. **(B)** Kaplan-Meier analysis of PFS in patients. **(C)** Kaplan-Meier analysis of OS in Patients. **(D)** ROC curve of tumor volume change rate in predicting DFS. **(E)** ROC curve of tumor volume change rate in predicting PFS. **(F)** ROC curve of tumor volume change rate in predicting OS.

Using ROC curve analysis with 3-year DFS as the endpoint, we identified an optimal prognostic cut-off of −50.62% for the tumor volume change rate by maximizing the Youden index (sensitivity = 0.733, specificity = 0.603). The results showed that patients with a tumor volume reduction rate ≥50.62% (i.e., volume change rate ≤−50.62%) had significantly better DFS (p = 0.001), PFS (p = 0.001) and OS (p = 0.029) than those with a reduction rate <50.62% (**Figure 4**). When this median cutoff was used as the criterion, the area under the ROC curve (AUC) of the tumor volume change rate for predicting 2-years DFS, PFS, and OS was 0.619, 0.632, and 0.657, respectively. The corresponding AUCs for predicting 3 DFS, PFS, and OS were 0.668, 0.661, and 0.660 (**Figure 4**).

### Multicenter validation and model performance evaluation of the tumor volume change rate

**Figure 5:**
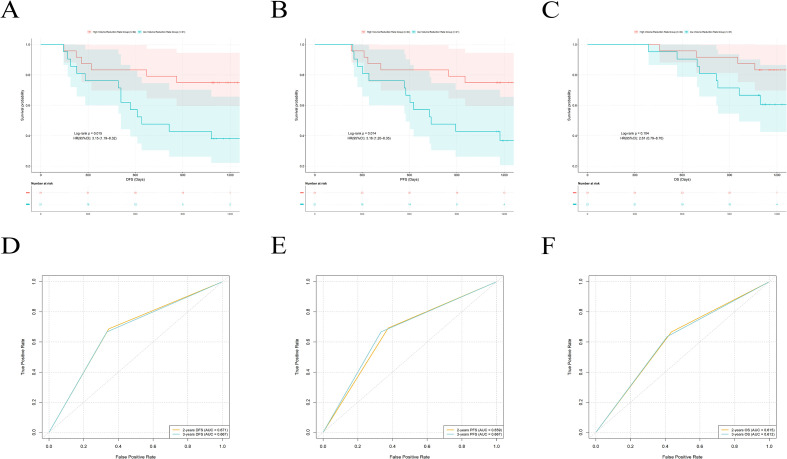
Kaplan-Meier survival analysis and roc curves in the external validation cohort. **(A)** Kaplan-Meier analysis of DFS in patients. **(B)** Kaplan-Meier analysis of PFS in patients stratified by high and low tumor volume change rate. **(C)** Kaplan-Meier analysis of OS in patients stratified by high and low tumor volume change rate. **(D)** ROC curve of tumor volume change rate in predicting DFS. **(E)** ROC curve of tumor volume change rate in predicting PFS. **(F)** ROC curve of tumor volume change rate in predicting OS.

We further validated the above findings using an external cohort of 45 patients from Yantai Yuhuangding Hospital. When patients were stratified by the same cutoff, those with a tumor volume reduction rate ≥50.62% again showed superior DFS and PFS compared to the group with a reduction rate <50.62%, successfully replicating the survival difference observed in the training cohort (**Figure 5**). However, we observed no statistically significant difference in OS between the two groups. This result may be related to the relatively limited number of events observed during the study. Additionally, the AUC of this cutoff value for predicting 2-years DFS, PFS, and OS in the validation cohort was 0.671, 0.659, and 0.615, respectively. The corresponding AUCs for predicting 3-years outcomes were 0.667, 0.667, and 0.612, which further confirms its predictive performance (**Figure 5**).

### An exploration of the timing of surgery following neoadjuvant therapy

**Figure 6:**
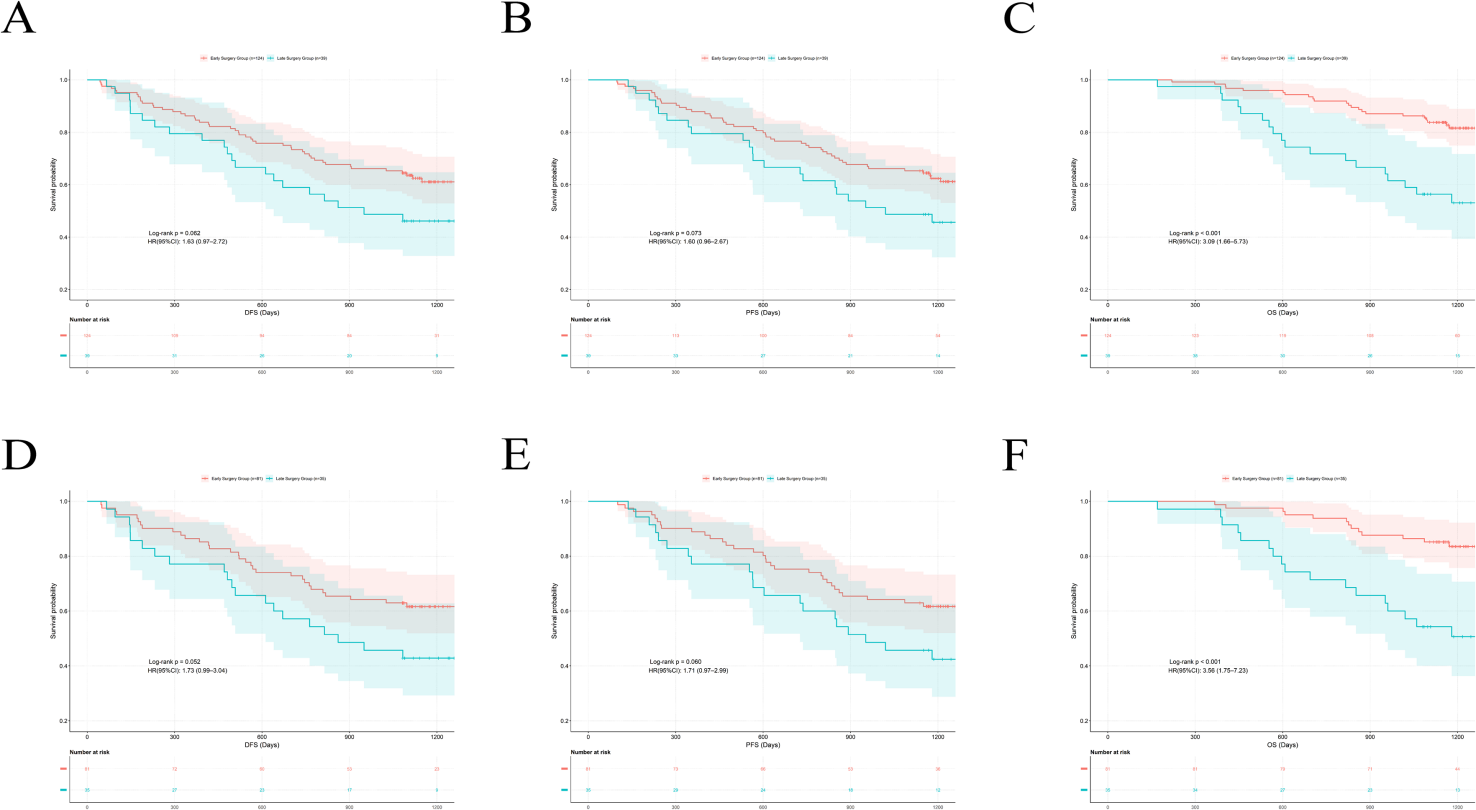
Kaplan-Meier survival analysis after PSM in early and late surgery groups. **(A)** Kaplan-Meier curve for DFS before PSM. **(B)** Kaplan-Meier curve for PFS before PSM. **(C)** Kaplan-Meier curve for OS before PSM. **(D)** Kaplan-Meier analysis of DFS after PSM. **(E)** Kaplan-Meier analysis of PFS after PSM. **(F)** Kaplan-Meier analysis of OS after PSM.

To evaluate the impact of the timing of surgery following neoadjuvant chemoimmunotherapy on patient outcomes, we conducted further analysis on the entire cohort. Patients were stratified into an early surgery group and a late surgery group based on whether the interval to surgery exceeded 6 weeks. After performing 1:3 PSM, results showed that the early surgery group exhibited a tendency toward longer DFS (p = 0.052) and PFS (p = 0.060), along with better OS (p < 0.001) (**Table 5** and **Figure 6**).

**Table 5:** Comparison of baseline characteristics between early and delayed surgery groups, before and After PSM.

Variable		Before PSM				After PSM			
		Early surgery (n=124)	Late surgery (n=39)	P	SMD	Early surgery (n=81)	Late surgery (n=35)	P	SMD
Volume Change Rate	≤-50.6%	61 (49.19)	19 (48.72)	0.959	-0.010	32 (39.51)	15 (42.86)	0.736	0.068
	>-50.6%	63 (50.81)	20 (51.28)		0.010	49 (60.49)	20 (57.14)		-0.068
Sex	female	17 (13.71)	6 (15.38)	0.793	0.046	9 (11.11)	5 (14.29)	0.864	0.091
	male	107 (86.29)	33 (84.62)		-0.046	72 (88.89)	30 (85.71)		-0.091
Age (years)	<60	46 (37.10)	15 (38.46)	0.878	0.028	30 (37.04)	15 (42.86)	0.555	0.118
	≥60	78 (62.90)	24 (61.54)		-0.028	51 (62.96)	20 (57.14)		-0.118
Kps	<90	99 (79.84)	28 (71.79)	0.291	-0.179	65 (80.25)	28 (80.00)	0.976	-0.006
	≥90	25 (20.16)	11 (28.21)		0.179	16 (19.75)	7 (20.00)		0.006
Tumor Location	Upper	19 (15.32)	9 (23.08)	0.263	0.184	15 (18.52)	8 (22.86)	0.591	0.103
	Middle and Lower	105 (84.68)	30 (76.92)		-0.184	66 (81.48)	27 (77.14)		-0.103
T stage	≤2	12 (9.68)	4 (10.26)	1.000	0.019	8 (9.88)	4 (11.43)	1.000	0.049
	>2	112 (90.32)	35 (89.74)		-0.019	73 (90.12)	31 (88.57)		-0.049
N stage	<2	89 (71.77)	29 (74.36)	0.753	0.059	63 (77.78)	27 (77.14)	0.940	-0.015
	≥2	35 (28.23)	10 (25.64)		-0.059	18 (22.22)	8 (22.86)		0.015
TRG	<2	65 (52.42)	21 (53.85)	0.876	0.029	44 (54.32)	18 (51.43)	0.774	-0.058
	≥2	59 (47.58)	18 (46.15)		-0.029	37 (45.68)	17 (48.57)		0.058
NICT cycles	2	90 (72.58)	29 (74.36)	0.827	0.041	61 (75.31)	26 (74.29)	0.907	-0.023
	>2	34 (27.42)	10 (25.64)		-0.041	20 (24.69)	9 (25.71)		0.023
Adjuvant therapy	No	29 (23.39)	14 (35.90)	0.122	0.261	22 (27.16)	10 (28.57)	0.876	0.031
	Yes	95 (76.61)	25 (64.10)		-0.261	59 (72.84)	25 (71.43)		-0.031

PSM, Propensity score matching; KPS, Karnofsky Performance Status; TRG, Tumor Regression Grade; NICT, Neoadjuvant Chemoimmunotherapy; Time Interval, Duration from NICT completion to surgery.

## Discussion

Through multicenter cohort validation, this study demonstrates that the rate of tumor volume change during neoadjuvant immunotherapy is significantly associated with patient survival. Compared with traditional RECIST-based unidimensional measurements, the three-dimensional volumetric analysis employed in this study captures dynamic changes in tumor burden more comprehensively and may have the potential to serve as a more sensitive imaging biomarker for response assessment in the immunotherapy setting. NCRT is the standard of care for resectable, locally advanced ESCC. However, its application is constrained by treatment-related toxicities, prompting the search for less toxic alternative strategies (11, 27). In recent years, immunotherapy has advanced from second-line to frontline treatment in advanced esophageal cancer, validating its potential and drawing increased attention to its application in locally advanced disease (28, 29). The use of NICT in locally advanced esophageal cancer remains a subject of debate. This study adds to the growing body of evidence supporting the efficacy of this combination strategy. The results showed that the neoadjuvant chemotherapy combined with immunotherapy regimen demonstrated significant efficacy, achieving a pCR rate of 31.3% and a 3-years OS rate of 79.1%. Following treatment, most patients showed a trend of tumor bed reduction, which effectively lowered the tumor burden. This created favorable conditions for reducing surgical difficulty and improving the rate of radical resection, further confirming the feasibility of this treatment approach. In addition, we found that patients receiving more than two cycles of NICT had significantly worse outcomes. While this may reflect a higher baseline tumor burden, the number of treatment cycles remained an independent prognostic factor after adjusting for T stage, N stage, and other clinicopathological variables. This association may be partly attributable to extended treatment delaying surgery — a view supported by our secondary analysis indicating (after PSM) that surgery performed within six weeks was associated with better outcomes. However, given the limited sample size of this study, this hypothesis requires further validation in prospective research.

Tumor volume has been established as a significant prognostic factor across various solid tumors (30, 31). Although the volumetric thresholds used vary across studies, multiple lines of evidence indicate that a significant reduction in primary tumor volume in esophageal cancer is generally associated with improved long-term survival, whereas a larger residual tumor volume conversely suggests a poorer prognosis (25, 32, 33).Nevertheless, in the field of esophageal cancer, particularly within the context of neoadjuvant immunotherapy, the impact of tumor volume and its dynamic changes on patient prognosis has not been systematically investigated. This study aimed to systematically evaluate the prognostic value of primary tumor volume and volumetric changes for long-term outcomes. The results showed that a reduction rate in tumor volume ≥50% during neoadjuvant therapy was significantly associated with an improved prognosis. This finding aligns with previous studies demonstrating that greater tumor regression correlates with more significant survival benefit (25). It is worth emphasizing that this study further validates the prognostic value of tumor volume changes in the context of immunotherapy, providing a basis for the broader application of this metric in novel treatment modalities. Furthermore, this study confirmed a significant correlation between the rate of tumor volume change following neoadjuvant immunotherapy and TRG, providing a pathological basis for the use of CT measurements in treatment efficacy assessment. Correlation analysis revealed a positive correlation between the degree of pathological response and the rate of volume change, suggesting a possible association between tumor killing effects at the microscopic level and macroscopic morphological changes (34, 35). However, it is important to recognize the inherent limitations of anatomical imaging in distinguishing residual tumor from treatment-related changes. Tumor volume reduction may result from various mechanisms, including tumor cell killing, treatment-induced fibrosis, necrosis, or resolution of inflammatory edema—each with different prognostic implications (36). Conversely, patients with stable disease or even slight enlargement on imaging may still exhibit significant pathological response. For instance, this study observed that 12 patients (7.4%) showed a slight increase in primary tumor volume following neoadjuvant immunotherapy. In the context of immunotherapy, this phenomenon warrants attention, as it may be partially attributable to “pseudo progression”—a temporary radiological volume increase caused by treatment-induced local lymphocytic infiltration, edema, or necrosis, rather than true tumor cell proliferation (37). Since all patients underwent curative surgery, we were able to conduct an in-depth analysis of these 12 patients through postoperative pathological examination. The results showed that 5 patients (41.7%) exhibited the paradoxical phenomenon of “imaging-detected volume increase but significant pathological response,” suggesting the possibility of pseudo progression. This finding indicates that, in clinical practice, tumor volume enlargement on imaging following immunotherapy should not be immediately interpreted as treatment failure; rather, careful evaluation is warranted to avoid prematurely discontinuing a potentially effective regimen. The remaining 7 patients (58.3%) had poor pathological responses (TRG 2–3), and their radiological enlargement was more consistent with true disease progression. This suggests that, in the overall study population, tumor volume enlargement detected by CT after neoadjuvant immunotherapy is still associated with poor treatment response and unfavorable prognosis in most cases. This imaging–pathology discrepancy suggests that tumor volume changes on imaging should be regarded as a “screening tool” rather than a “diagnostic tool” for assessing treatment efficacy. For patients with poor radiological response but good clinical status, the possibility of pseudo progression should be considered, and pathological verification should be actively pursued (37–39). Conversely, for patients with significant radiological remission, the possibility of residual viable tumor cells cannot be completely ruled out, and decisions regarding postoperative adjuvant therapy should still be guided by pathological assessment. These findings further highlight the irreplaceable role of pathological evaluation in assessing disease response, while also revealing the limitations of relying solely on CT morphological indicators for efficacy assessment in esophageal cancer patients receiving neoadjuvant immunotherapy. Future research should focus on integrating CT volumetric measurements with functional imaging (e.g., PET-CT, diffusion-weighted MRI) or liquid biopsy (e.g., dynamic monitoring of circulating tumor DNA) to establish a multimodal, multidimensional efficacy evaluation system. Such an approach may enable real-time, precise differentiation between residual tumor and treatment-related changes during therapy, thereby providing a more reliable basis for personalized treatment decisions.

In this study, 44 patients (27.0%) received ≥2 cycles of neoadjuvant therapy. The number of cycles was determined primarily based on the following clinical decision-making logic: For patients with a high baseline tumor burden (e.g., stage T4 or N+), multidisciplinary discussions tended to favor additional cycles to achieve greater tumor regression; conversely, for patients whose imaging assessments showed stable disease during treatment, the clinical team might recommend continuing the original regimen to pursue deeper remission, thereby creating more favorable conditions for subsequent surgery. Therefore, the number of treatment cycles is correlated with baseline tumor burden—we also observed a positive correlation between baseline tumor volume and the number of cycles (r = 0.186, P = 0.044), confirming that the selection of cycle numbers was a non-random decision based on clinical necessity. In other words, differences in the number of treatment cycles essentially reflect a combination of patients’ baseline characteristics, treatment response, and treatment tolerance, some of which are themselves important predictors of poor prognosis. Although we attempted to adjust for known confounding factors (such as TNM staging) in multivariate analysis, limitations in sample size and the retrospective study design prevented us from fully correcting for all potential confounders related to the selection of treatment cycles. Therefore, conclusions regarding the number of treatment cycles should be regarded as exploratory, and interpretation of their association with prognosis requires particular caution: one cannot simply infer that “more cycles” lead to “better or worse prognosis,” as the number of cycles is more likely a marker of the patient’s clinical status and treatment course rather than an independent determinant of prognosis. Among the 39 patients who underwent surgery more than 6 weeks after the initially scheduled date, the reasons for delay were heterogeneous. Specifically, 2 cases were postponed due to COVID-19 infection, 1 case due to immune-mediated myocardial injury, and the remaining patients due to delayed hospital readmission for personal reasons. Notably, the vast majority (92.3%) of delays stemmed from personal factors, such as family matters, transportation difficulties, or individual preference. Such factors are typically not directly associated with the patient’s tumor biology or treatment response, providing a natural observational setting to assess the impact of “delay itself” on prognosis. If these patients are comparable to those who underwent surgery on schedule in terms of baseline characteristics and pathological response, the observed survival differences may more closely reflect the true effect of surgical delay. Compared with traditional NCRT, NICT has a lower incidence of adverse reactions, which are primarily mild; therefore, it has less impact on the normal progression of surgery (22). However, a small proportion of delays were still associated with treatment-related complications and external uncontrollable factors (the COVID-19 pandemic). Adverse reactions associated with immune checkpoint inhibitors are themselves one of the factors directly contributing to a poorer prognosis (40, 41). It is important to emphasize that even if most delays stem from personal reasons, such factors may still be associated with unmeasured confounders such as socioeconomic status and health literacy, which may themselves influence prognosis. Therefore, although the heterogeneity of delay causes provides some insight into the direction of causality, confounding bias cannot be completely ruled out. Mechanistically, tumor cells may re-enter a phase of proliferation after the completion of neoadjuvant therapy; delaying surgery may provide a time window for the recovery and expansion of residual tumor cells, thereby increasing the risk of local recurrence (42). Micro metastases may progress to clinically detectable metastatic disease during the waiting period, transforming a potentially curable localized lesion into disseminated disease (43, 44). Furthermore, the antitumor immune response induced by neoadjuvant immunotherapy may gradually wane after treatment ends; delaying surgery may cause patients to miss the optimal window for the full realization of immune effects, thereby depriving patients who could have benefited from early surgical intervention of the best treatment opportunity (45, 46).

This study found that, after propensity score matching, patients who underwent early surgery (≤6 weeks) had a significant OS benefit, whereas DFS and PFS showed only a trend toward improvement. This discrepancy does not imply that early surgery has limited clinical value for DFS or PFS; rather, it may stem from differences in endpoint definitions, recurrence patterns, and the nature of salvage therapy. The underlying mechanism is likely closely related to the more aggressive recurrence patterns induced by delayed surgery. First, the definitions of the endpoints warrant consideration. OS, measured from the initiation of neoadjuvant immunotherapy to death from any cause, is a classic and robust indicator of survival benefit, less susceptible to information bias (47). Second, as mentioned above, delaying surgery may be associated with a higher risk of tumor recurrence. Tumor clones surviving under immunotherapy pressure may undergo immune editing and acquire a more aggressive phenotype (e.g., enhanced proliferation, increased metastatic potential, and treatment resistance) (48, 49). In other words, when patients in the delayed surgery group experience recurrence, the recurrence may be more aggressive (e.g., a higher proportion of distant metastases), progress more rapidly, and respond poorly to salvage therapy, resulting in significantly shorter post-recurrence survival and ultimately a significant OS difference, despite a relatively modest difference in time to first recurrence (DFS/PFS). From the perspective of salvage therapy, local recurrence can often be managed with radiotherapy or repeat surgery, potentially extending post-recurrence survival. In contrast, distant metastases are generally incurable and directly increase the risk of death. This difference may explain why OS—but not DFS or PFS—showed a statistically significant difference between groups. It should be noted that in this study, both before and after PSM, the proportion of patients in the delayed surgery group who developed distant metastasis (including isolated distant metastasis and concurrent local and distant recurrence) was numerically higher than that in the early surgery group, although this difference did not reach statistical significance (before matching: 61.90% vs. 48.94%, P = 0.322; after matching: 65.00% vs. 45.16%, P = 0.166). This may be related to the limited sample size and the small number of events, and warrants further validation in larger cohorts. This hypothesis is partially supported by previous research. For instance, studies have shown that patients who underwent early surgery had significantly higher densities of CD8^+^ T cells in the tumor microenvironment compared to those with delayed surgery, suggesting that a prolonged preoperative interval may exacerbate tissue fibrosis, impede T-cell infiltration, and increase T-cell exhaustion, thereby enhancing tumor aggressiveness (42). In summary, we propose that delayed surgery may exert a significant impact on long-term survival by inducing a more aggressive recurrence pattern. This suggests that when evaluating the optimal timing of surgery following neoadjuvant immunotherapy, attention should be paid not only to the time to first recurrence but also to the nature of post-recurrence disease progression and its potential impact on overall survival. Large-scale prospective studies are needed to further validate this hypothesis.

In clinical practice, imaging-based assessment of treatment response is indispensable in oncology. The most widely used criteria for evaluating solid tumors—Response Evaluation Criteria in Solid Tumors (RECIST)—rely on clearly measurable target lesions (25). However, primary lesions of the digestive tract are typically not considered measurable due to the considerable difficulty in obtaining stable measurements across multiple examinations (25). For esophageal cancer, the “longest diameter” of the tumor on CT images often proves challenging to define accurately following multiple cycles of treatment (50). Currently, there remains a lack of unified criteria for evaluating tumor contours following treatment. Compared to single-layer esophageal wall thickness, the volumetric measurement of the entire tumor segment may serve as a more robust and accurate metric, which could also enhance inter-observer consistency and measurement reproducibility.

This study has several limitations. First, this study excluded changes in regional lymph node volume following neoadjuvant therapy from the prognostic analysis, primarily because the shortest diameter of the lymph nodes in some patients was less than 10 mm at baseline or after treatment. This rendered accurate and reproducible quantitative delineation and measurement on CT images challenging. This omission may have potential implications for prognostic assessment. On one hand, ypN staging is a well-established prognostic factor in esophageal cancer, and the absence of lymph node volume change data limits the comprehensiveness of current prognostic models. On the other hand, tumor response to treatment may differ between the primary lesion and regional lymph nodes. Some patients may show significant regression of the primary tumor while residual disease persists in the lymph nodes, or vice versa. Such heterogeneity in response may introduce bias in prognostic evaluations for certain individuals and hinder the comprehensive reflection of the overall efficacy of neoadjuvant chemoradiotherapy on the “primary tumor–lymph node” complex. To address these limitations, future research should incorporate modalities such as functional imaging to enhance the accuracy of assessing lymph node response. In addition, joint analyses of volumetric changes in both the primary tumor and lymph nodes are warranted to develop an integrated prognostic model that incorporates information from both components, thereby improving the precision of post-neoadjuvant therapy prognostic assessment. Second, the sample size was relatively small—particularly in the external validation cohort, which included only 45 patients—limiting the statistical power. Moreover, the selection of chemotherapy and immunotherapy regimens was heterogeneous, and the prognostic impact of this heterogeneity requires validation in larger cohorts. Furthermore, the current follow-up period is relatively short, and complete survival data are not yet mature. Therefore, the precise survival benefit of NICT remains difficult to accurately assess and requires longer follow-up to clarify its long-term efficacy. With future updates of survival data, the relationship between the rate of tumor volume change rate and long-term prognosis may be more clearly elucidated. Despite these limitations, the findings of this study confirm the important value of CT in evaluating neoadjuvant chemoimmunotherapy for esophageal squamous cell carcinoma. Particularly in medical centers not equipped with PET/CT, the tumor volume change rate can serve as a reliable indicator to distinguish between significant and non-significant responders, thereby informing subsequent treatment decisions. This study transforms routine imaging into a dynamic, quantifiable clinical decision-support tool. By assessing the degree of treatment response early during therapy, it provides clinicians with a critical decision window to promptly adjust treatment for high-risk patients with predicted poor prognosis, thereby advancing precision therapy and potentially improving patient survival.

## Conclusion

By analyzing CT imaging changes in esophageal cancer patients during neoadjuvant chemoimmunotherapy, this study confirms a significant correlation between the rate of tumor volume change and patient prognosis. This finding was successfully validated in an independent multi-center cohort, demonstrating its robustness and generalizability. Furthermore, we recommend that surgical treatment be performed within 6 weeks of completing neoadjuvant therapy.

## Data Availability

The original contributions presented in the study are included in the article/supplementary material. Further inquiries can be directed to the corresponding author.
